# Sugar accumulation and growth of lettuce exposed to different lighting modes of red and blue LED light

**DOI:** 10.1038/s41598-019-43498-8

**Published:** 2019-05-06

**Authors:** Xiao-li Chen, Li-chun Wang, Tao Li, Qi-chang Yang, Wen-zhong Guo

**Affiliations:** 1 Beijing Research Center of Intelligent Equipment for Agriculture, Beijing Academy of Agriculture and Forestry Sciences, Beijing, 100097 China; 2 Institute of Environment and Sustainable Development in Agriculture, Chinese Academy of Agricultural Sciences, Beijing, 100081 China; 3 Key Laboratory of Urban Agriculture (North China), Ministry of Agriculture and Rural Affairs, Beijing, China

**Keywords:** Plant physiology, Light responses

## Abstract

The present study evaluated the growth response and sugar accumulation of lettuce exposed to different lighting modes of red and blue LED light based on the same daily light integral (7.49 μmol·m^−2^). Six lighting treatments were performed, that were monochromatic red light (R), monochromatic blue light (B), simultaneous red and blue light as the control (RB, R:B = 1:1), mixed modes of R, B and RB (R/RB/B, 4 h R to 4 h RB and then 4 h B), and alternating red and blue light with alternating intervals of 4 h and 1 h respectively recorded as R/B(4 h) and R/B(1 h). The Results showed that different irradiation modes led to obvious morphological changes in lettuce. Among all the treatments, the highest fresh and dry weight of lettuce shoot were both detected with R/B(1 h), significantly higher than the other treatments. Compared with plants treated with RB, the contents of fructose, glucose, crude fiber as well as the total sweetness index (TSI) of lettuce were significantly enhanced by R treatment; meanwhile, monochromatic R significantly promoted the activities of sucrose degrading enzymes such as acid invertase (AI) and neutral invertase (NI), while obviously reduced the activity of sucrose synthesizing enzyme (SPS). Additionally. The highest contents of sucrose and starch accompanied with the strongest activity of SPS were detected in plants treated with R/B(1 h). The alternating treatments R/B(4 h) and R/B(1 h) inhibited the activity of SS, while enhanced that of SPS compared with the other treatments, indicating that different light environment might influence sugar compositions via regulating the activities of sucrose metabolism enzymes. On the whole, R/B(1 h) was the optimal lighting strategy in terms of lettuce yield, taste and energy use efficiency in the present study.

## Introduction

Light drives photosynthesis as energy source, and controls plenty of plant physiological processes as signals^1,2^. Light control is one of the important aspects in controlled-environment agriculture (CEA) especially for closed vertical farming systems where artificial lamps are the only light source for plant growth. Plant responses can be triggered by changes in light intensity, light quality and photoperiod, among which light quality acts much more complicated effects on plant morphology and physiology. Light quality affects gene expression through initiating the signaling cascade of photoreceptors like phytochromes, cryptochromes and phototropins^3,4^. As crucial light spectrum for plant growth, red light (R) and blue light (B) are efficiently absorbed by chlorophyll a and b, and have the largest impacts on seed germination, seedling de-etiolation, flowering time, leaf development, stomatal opening, chloroplast accumulation, anthocyanin biosynthesis and circadian clock^5–7^.

Soluble sugar and crude fiber are carbohydrates affecting the sweetness and crispness of lettuce^8–10^. Soluble sugar in lettuce is mainly consisted of fructose, glucose and sucrose, and the total sweetness index (TSI) is determined by the sweetness coefficient and concentration of each soluble sugar^11,12^. It was reported that R and B had impacts on carbohydrate accumulations and compositions which determine both flavor and quality of vegetables^13^. Monochromatic R demonstrated significant promoting effects on soluble sugar and starch accumulations in upland cotton plantles and rapeseed relative to FL (fluorescent light), RB or B^14,15^. Li^16^ also reported that monochromatic R significantly increased the contents of fructose and glucose in tomato seedling leaves compared with W (white LED light), B or RB. Choi^17^ reported that monochromatic B inhibited the sucrose formation in strawberry compared with those under R or RB; in contrast, Fan^18^ reported that monochromatic B promoted soluble sugar accumulation in non-heading Chinese cabbage compared with W, R or RB. The combination of R and B significantly enhanced total carbohydrate, starch and sucrose accumulation in tomato seedling leaves compared with W, R or B^16^. Chen^19^ proposed that the influence of intermittent RB on carbohydrate amount in lettuce were related to the light/dark circles or intermittent frequencies. However, the light environment composed of R or B in studies mentioned above was all consistent, dynamic light environment composed of R or B has scarcely been concerned.

In dynamic light environment, light intensity or light quality or both regularly changes during a light period, rather that being consistent or unchangeable. According to some studies, dynamic irradiation of R and B might bring different results compared with consistent irradiation. For example, Yamada^20^ proposed that dry weight of sweetpotato in stepwise photosynthetic photon flux (PPF) increased by 10% relative to that in constant PPF on the premise of the same light integral during the experimental period. Hoffmann^21^ claimed that blue light with alternating low and high intensity promoted the anthocyanin synthesis and flavonol accumulation in pepper leaves. Shimokawa^22^ proposed that alternating R/B light caused increase in lettuce yield, while Jao and Fang^23^ found that the biomass of potato plantlets illuminated with alternating R/B light was significantly decreased. The effects of dynamic RB light environment on sugar accumulation in lettuce have scarcely been concerned. It is necessary to conduct the comparisons between the consistent light environment and dynamic light environment for the comprehensive exploration.

In addition, the activity of sucrose metabolism-related enzymes is an important factor in sugar metabolism, and carbohydrate metabolism regulation has been widely studied based on the sucrose metabolism-related enzymes^24–26^. The crucial enzymes associated with sucrose metabolism and carbohydrate composition are sucrose synthase(SS), invertase(AI, NI) and sucrose phosphate synthase (SPS)^27,28^. Li^16^ studied the effects of light quality on the activities of the above mentioned enzymes in tomato seedling leaves, and found that RB (especially 3R1B) promoted SS activity compared with monochromatic R, B or W; monochromatic R significantly increased the activities of AI and NI while reduced that of SPS in comparison with RB or W. Nevertheless, the influence of dynamic RB light environment on the sucrose metabolism-related enzymes have scarcely been studied.

The purpose of the study was to compare the effects of consistent RB light environment and dynamic RB light environment on sugar accumulation and growth of lettuce based on the same light quantum. Lettuce biomass, morphology, the amounts of sucrose, fructose, glucose, starch, crude fiber and the enzyme activities related to sucrose metabolism were studied to determine the functions of different light environment. The study will help for a deeper understanding of the relationship between red and blue light when acting on plants; the results are expected to optimize the application modes and lighting strategies of red and blue light in a horticultural production system.

## Methodology

### Experimental conditions

The experiment was carried out in a closed plant factory in Beijing Academy of Agriculture and Forestry Sciences (BAAFS). Lettuce seeds (*Lactuca sativa* L.) were sowed in sponges for seedling cultivation. After 14 days, seedlings were transplanted into hydroponic boxes under different light environments The environmental conditions in the plant factory were 23 ± 2 °C, 600 μmol·mol^−1^ CO_2_ level and 65% relative humidity (RH). 15 plants spaced 20 cm apart were planted in each hydroponic box (100 × 60 × 10 cm). The pH and EC of Hoagland’s solution^29^ were kept at 5.8–6.0 and 0.11–0.12 S·m^−1^ respectively. The plants were irradiated with six different light treatments for 40 days and harvested at 54 days after sowing (DAS).

### Light treatments

**Figure 1:**
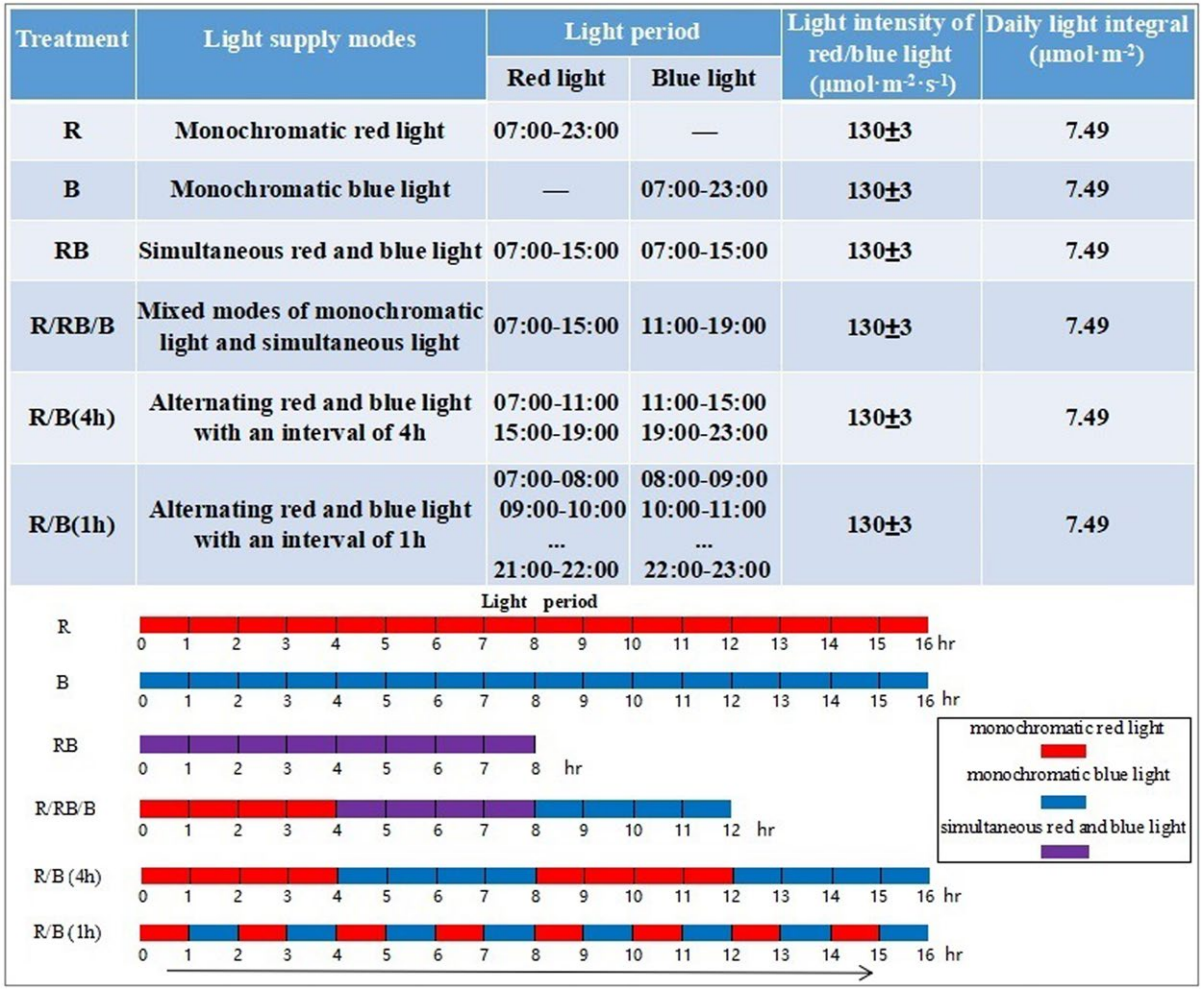
The irradiation modes of LED light over a 24-hour period in different treatments. Treatment R provided monochromatic red light for 16 h while treatment B provided monochromatic blue light in the same period. Treatment RB provided simultaneous red and blue light for 8 h. Light in treatment R/RB/B switched from monochromatic red (lasted for 4 h) to simultaneous red and blue (lasted for 4 h) and then to monochromatic blue (lasted for 4 h). The alternating irradiation provided by R/B(4 h) was 4 h:4 h (i.e., 4 h of red light and 4 h of blue light during a 16 h photoperiod), and by R/B(1 h) was 1 h:1 h (i.e., 1 h of red light and 1 h of blue light during a 16 h photoperiod).

Two-color LED panels emitting R and B with peak wavelength of respectively 660 nm and 450 nm were used as light sources in the study. The photosynthetic photon flux density (PPFD) of R and B could be regulated by adjusting the electric current of DC power supply individually, and the alternating intervals of R and B could also be individually controlled by the built-in timing switches. The PPFD of R and B measured at plant canopy level were both 130 ± 3 μmol·m^−2^·s^−1^ in all treatments. As shown in Fig. 1, six lighting modes included monochromatic red light (R), monochromatic blue light (B), simultaneous red and blue light as the control (RB, R:B = 1:1), mixed modes of R, B and RB (R/RB/B, 4 h R to 4 h RB and then 4 h B), and alternating red and blue light with alternating intervals of 4 h and 1 h respectively recorded as R/B(4 h) and R/B(1 h). The daily light integral in all treatments was the same 7.49 μmol·m^−2^. In addition, the number of red or blue light quantum was the same among the four treatments RB, R/RB/B, R/B(4 h) and R/B(1 h) over the whole growth period, so was the total electric energy consumption by LEDs.

### Sampling and sample processing

Plant morphology was described based on three representative plants taken from each treatment at harvest (54 DAS). Fresh lettuce samples were taken for fresh weight (FW) and pigment content measurements. Parts of the harvested fresh samples were oven-dried at 70 °C for 48 h for dry weight (DW) and sugar content measurements. Additionally, some fresh samples were frozen with liquid nitrogen and stored in a super cold refrigerator at −80 °C for enzyme activity determination. Three plants randomly taken from each hydroponic box were regarded as a repetition for index measurements, and there were three repetitions in each treatment.

### Determination of pigments

0.2 g samples of the fresh mature leaves were ground and washed with 80% acetone until the samples turned white. The solution was collected and filtered and the filtrate was supplemented with distilled water to a total volume of 100 ml. A spectrophotometer (TU-1810s, PERSEE, China) was used to measure the absorbance of the extraction at 470 nm, 645 nm, and 663 nm respectively. The contents of chlorophyll (Chl) and carotenoid (Car) were determined according to the equations as the follows^30^:

$${\rm{Chl}}\,{\rm{a}}\,(\mathrm{mg}/g)=\tfrac{(12.72\,\times \,{{\rm{OD}}}_{663}-2.59\,\times \,{{\rm{OD}}}_{645}){\rm{V}}}{1000\,{\rm{W}}}$$

$${\rm{Chl}}\,{\rm{b}}\,(\mathrm{mg}/g)=\tfrac{(22.88\,\times \,{{\rm{OD}}}_{645}-4.67\,\times \,{{\rm{OD}}}_{663}){\rm{V}}}{1000\,{\rm{W}}}$$

$${\rm{Car}}\,(\mathrm{mg}/g)=\tfrac{((1000\,\times \,{{\rm{OD}}}_{470}-3.27\,\times \,{\rm{Chl}}.{\rm{a}}-104\,\times \,{\rm{Chl}}{\rm{.b}})\,/\,229){\rm{V}}}{1000\,{\rm{W}}}$$

V and W in the equation respectively mean the volume of the extract and the fresh weight of the sample.

### Determination of soluble sugar, starch and crude fiber

1.0 g lettuce shoot sample (DW) mixed with 5 ml 80% (v/v) ethanol was placed in a 80 °C water bath for 30 min and then centrifuged at 12,000**×** g for 10 min. The supernatant and precipitate was respectively collected for soluble sugar and starch measurements. The residues left after evaporating the supernatant at 85 °C was mixed with 20 ml distilled water and passed through 0.45 μm microporous membrane. The contents of fructose, glucose and sucrose were determined via the HPLC system^19^ based on the corresponding standards^31^. The total sweetness index (TSI) determined by the concentration and sweetness coefficient of each soluble sugar was calculated using the equation^12^:

$${\rm{TSI}}=1.50\times {\rm{fructose}}+0.76\times {\rm{glucose}}+1.00\times {\rm{sucrose}}.$$

3 ml deionized water was added into the precipitate collected for starch measurement and boiled for 15 min. The cooling precipitate mixed with 2 ml 30% (v/v) HClO_4_ was agitated and diluted to a total volume of 10 ml with distilled water. New supernatant was collected after centrifuging the solution at 12,000× g for 10 min. The starch content was determined accrording to the glucose liberated in the supernatant^19,32^. Dry residue collected after digesting the lettuce shoot sample with 1.25% (v/v) sodium hydroxide and 1.25% (v/v) sulphuric acid was ignited, and the crude fiber content was determined from the loss in weight and calculated via the following equation^33^:

$${\rm{Fiber}}( \% )=\tfrac{{\rm{loss}}\,{\rm{of}}\,{\rm{weight}}\,{\rm{on}}\,{\rm{ignition}}}{{\rm{weight}}\,{\rm{of}}\,{\rm{sample}}\,{\rm{used}}}\times 100$$

### Determination of enzyme activities

1.0 g frozen samples were mixed with liquid nitrogen and ground in a cooled mortar with 5 ml of 0.1 M Tris-HCl buffer as described by Chen^19^. After centrifuging the extract at 10,000× g for 20 min (at 2 °C), Tris-HCl buffer mentioned above was diluted 5 times and used to dialyse the supernatant. After dialysis, the supernatant was used for the determination of sucrose metabolism enzyme activities. The amount of reducing sugar released from sucrose was measured to assay the activities of sucrose synthase (SS, E.C.2.4.1.13), acid invertase (AI, E.C.3.2.1.26) and neutral invertases (NI, E.C.3.2.1.26) according to the method described by Gordon^34^ and King^35^. The amount of the produced sucrose was measured to assay the activity of sucrose phosphate synthase (SPS, E.C.2.4.1.14) as described by Lowell^36^ and Sun^37^.

### Statistical analysis

Statistical analysis was conducted with SPSS 11.0 software (SPSS Inc., Chicago, USA). Significance among the six treatments were performed by Tukey’s multiple range test at the 0.05 significance level.

## Results and Analysis

### Growth characteristics and biomass

**Figure 2:**
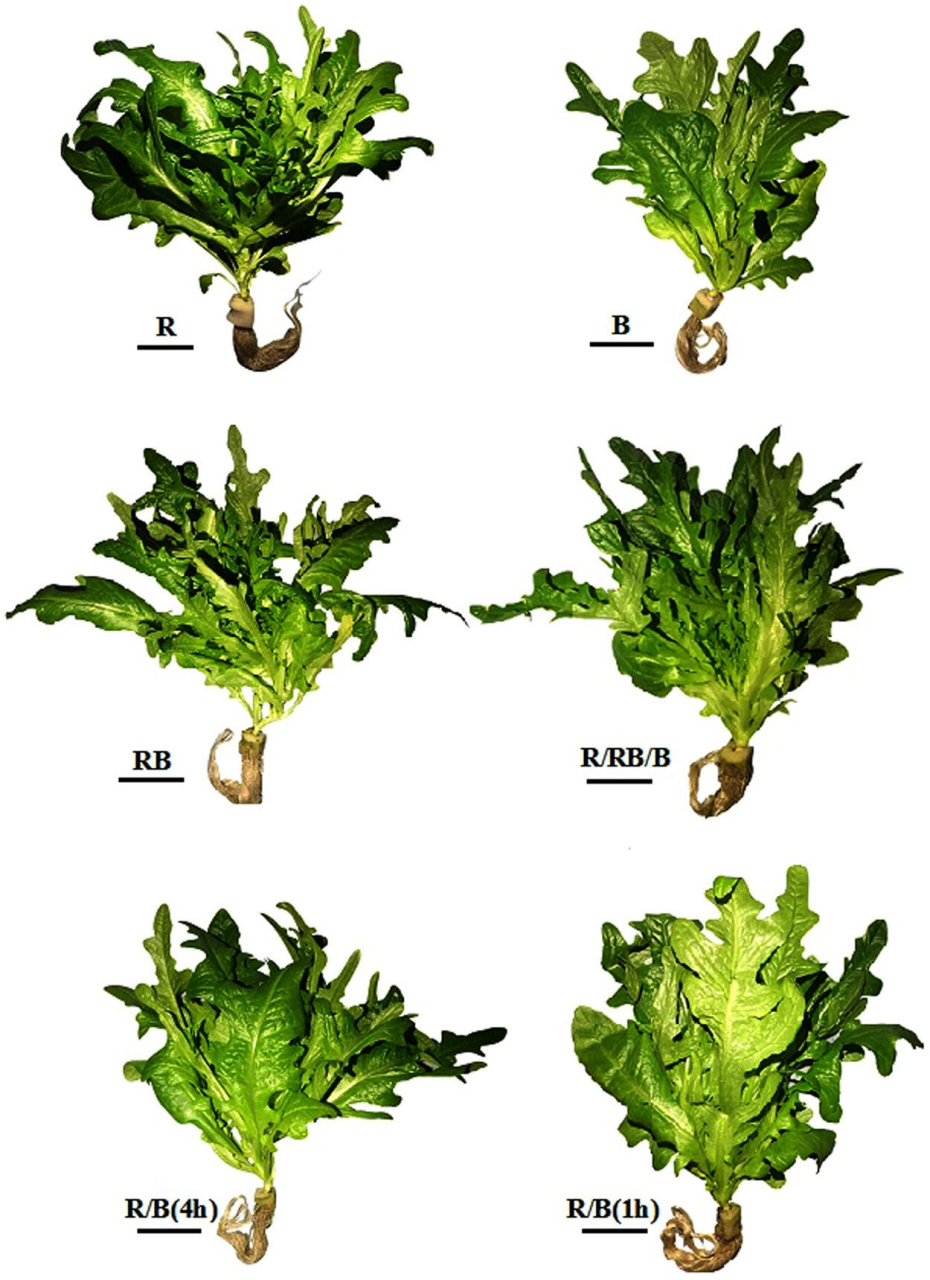
Morphology of lettuce (at harvest) planted in varied light treatments. The black bars indicate 5 cm. R: monochromatic red light for 16 h over a 16-hour photoperiod; B: monochromatic blue light for 16 h over a 16-hour photoperiod; RB: simultaneous red and blue light for 8 h over a 24-hour period; R/RB/B: light switching from monochromatic red (lasted for 4 h) to simultaneous red and blue (lasted for 4 h) and then to monochromatic blue (lasted for 4 h) over a 24-hour period; R/B(4 h): alternating red and blue light with an interval of 4 h over a 16-hour photoperiod; R/B(1 h): alternating red and blue light with an interval of 1 h over a 16-hour photoperiod.

As shown in Fig. 2, plants under pure R and B displayed very different morphology, plants with R treatment looked dense with narrow and tortuous leaves, and no excessive elongation was observed; while plants with B treatment were short with hypertrophic and thick leaves. Despite the same daily integral and electric energy consumption, the sparsest plant architecture with obvious stem elongation was detected under RB treatment, while vigorous and compact plant morphology were obtained in R/B(1 h) and R/RB/B treatments. Chen^38^ reported that monochromatic R tended to result in excessive elongation during the seedling stage of lettuce. Obvious stem elongation of lettuce at harvest detected under RB treatment in the present study indicated that simultaneous R and B with a relatively short photoperiod (8 h in the present study) might also lead to stem elongation of lettuce, thus, stem elongation may not be only associated with light quality and the growth period, but also with the light photoperiod.

As shown in Table 1, the fresh and dry weights of lettuce shoot were both the highest under R/B(1 h) treatment, reaching significant level compared with any other treatment in the present study. Compared with RB, the fresh weight of lettuce shoot treated with R and R/RB/B was significantly increased by approximately 10.5%, on the contrary, B and R/B (4 h) decreased the fresh weight of lettuce shoot by respectively by 9.4% and 17.3%. It indicated that dynamic light environment did have the potential to enhance lettuce yield.

**Table 1:** Biomass and growth characters of lettuce at harvest (54 DAS).

Light treatment	Fresh weight (g)		Dry weight (g)		Plant height (mm)	Stem diameter (mm)	Leaf number
	Shoot	Root	Shoot	Root			
R	83.03b	8.63a	3.75b	0.37a	264.13a	8.29b	21ab
B	61.71d	6.12c	2.96c	0.29b	210.63c	7.41c	18c
RB	74.66c	7.60b	3.04bc	0.40a	227.65b	7.39c	20b
R/RB/B	82.02b	8.62a	3.88b	0.38a	251.03ab	7.95bc	22a
R/B(4 h)	67.65cd	7.03b	3.55b	0.38a	229.10b	7.02c	20b
R/B(1 h)	93.33a	8.00a	4.36a	0.36a	255.64ab	9.25a	22a

Lower-case letters after the same parameter indicate significant difference at the 0.05 level by Tukey’s test, n = 3.

R: monochromatic red light for 16 h over a 16-hour photoperiod; B: monochromatic blue light for 16 h over a 16-hour photoperiod; RB: simultaneous red and blue light for 8 h over a 24-hour period; R/RB/B: light switching from monochromatic red (lasted for 4 h) to simultaneous red and blue (lasted for 4 h) and then to monochromatic blue (lasted for 4 h) over a 24-hour period; R/B(4 h): alternating red and blue light with an interval of 4 h over a 16-hour photoperiod; R/B(1 h): alternating red and blue light with an interval of 1 h over a 16-hour photoperiod.

### Pigment content

**Figure 3:**
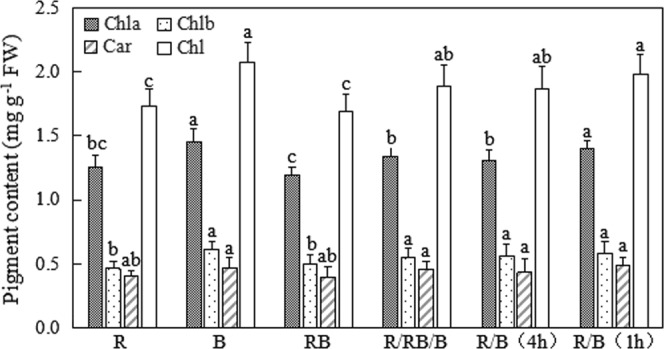
The pigment contents of lettuce (at harvest) planted in varied light treatments. Lower-case letters with the same parameter indicate significant difference at the 0.05 level by Tukey’s test, n = 3. The bars indicate the standard errors. Chl: chlorophyll; Chla: chlorophyll a; Chlb: chlorophyll b; Car:carotenoid. R: monochromatic red light for 16 h over a 16-hour photoperiod; B: monochromatic blue light for 16 h over a 16-hour photoperiod; RB: simultaneous red and blue light for 8 h over a 24-hour period; R/RB/B: light switching from monochromatic red (lasted for 4 h) to simultaneous red and blue (lasted for 4 h) and then to monochromatic blue (lasted for 4 h) over a 24-hour period; R/B(4 h): alternating red and blue light with an interval of 4 h over a 16-hour photoperiod; R/B(1 h): alternating red and blue light with an interval of 1 h over a 16-hour photoperiod.

The contents of chlorophyll and carotenoid in lettuce planted in varied light environment were presented in Fig. 3. The general trend demonstrated that B and R/B(1 h) treatments led to higher pigment contents of lettuce. The dynamic light environment in R/RB/B, R/B(4 h) and R/B(1 h) treatments tended to increase Chl a and Chl b contents of lettuce compared with the consistent light environment in RB treatment. No significant difference was observed in Car content among all the treatments.

### The contents of fructose, glucose and sucrose

**Figure 4:**
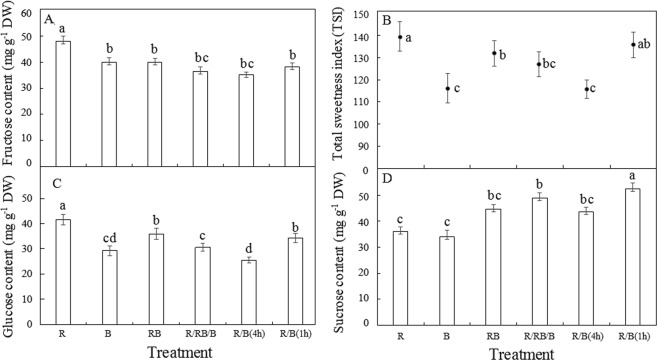
The contents of each soluble sugar and the TSI of lettuce (at harvest) planted in varied light treatments. Lower-case letters with the same parameter indicate significant difference at the 0.05 level by Tukey’s test, n = 3. The bars indicate the standard errors. R: monochromatic red light for 16 h over a 16-hour photoperiod; B: monochromatic blue light for 16 h over a 16-hour photoperiod; RB: simultaneous red and blue light for 8 h over a 24-hour period; R/RB/B: light switching from monochromatic red (lasted for 4 h) to simultaneous red and blue (lasted for 4 h) and then to monochromatic blue (lasted for 4 h) over a 24-hour period; R/B(4 h): alternating red and blue light with an interval of 4 h over a 16-hour photoperiod; R/B(1 h): alternating red and blue light with an interval of 1 h over a 16-hour photoperiod.

The soluble sugar in lettuce is mainly composed by sucrose, glucose and fructose, among which, fructose has the highest sweetness, and the amount of fructose and glucose is summed as hexose. As shown in Fig. 4, the contents of fructose and glucose in lettuce treated with monochromatic R were both significantly higher than that in lettuce treated with the other treatments (p ≤ 0.05), indicating that monochromatic R might stimulate the accumulation of hexose in lettuce. Compared with the consistent light environment in RB treatment, the dynamic light environment in R/RB/B, R/B(4 h) and R/B(1 h) tended to reduce the contents of fructose and glucose in lettuce to different degrees, suggesting that the dynamic light environment in the current study had no positive effects on the hexose accumulation in lettuce. In contrast, sucrose content in lettuce was significantly enhanced by R/B(1 h) compared with the other treatments, while obviously decreased by monochromatic light in R or B treatment. Lettuce with the highest TSI was observed under R and R/B(1 h) treatments, and no significant difference was detected between the two treatments for TSI.

### Enzyme activity

**Figure 5:**
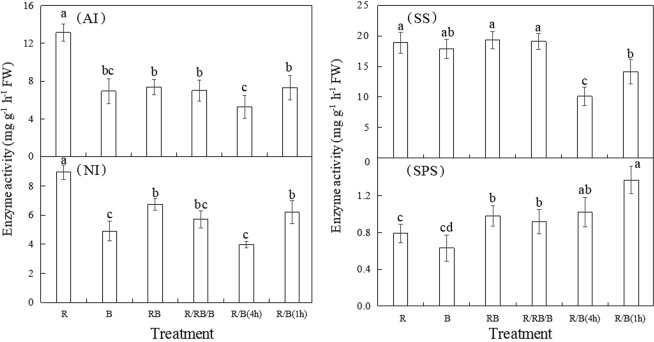
The activities of enzymes involved in sucrose metabolism of lettuce (at harvest) planted in varied light treatments. Lower-case letters with the same parameter indicate significant difference at the 0.05 level by Tukey’s test, n = 3. The bars indicate the standard errors. AI:acid invertases; NI:neutral invertases; SS:sucrose synthase (cleavage); SPS:sucrose phosphate synthase. R: monochromatic red light for 16 h over a 16-hour photoperiod; B: monochromatic blue light for 16 h over a 16-hour photoperiod; RB: simultaneous red and blue light for 8 h over a 24-hour period; R/RB/B: light switching from monochromatic red (lasted for 4 h) to simultaneous red and blue (lasted for 4 h) and then to monochromatic blue (lasted for 4 h) over a 24-hour period; R/B(4 h): alternating red and blue light with an interval of 4 h over a 16-hour photoperiod; R/B(1 h): alternating red and blue light with an interval of 1 h over a 16-hour photoperiod.

The activities of enzymes related to sucrose metabolism were presented in Fig. 5. The activity of invertase including AI and NI in lettuce was significantly promoted by monochromatic R comparing with the other treatments. It has been reported that SS in lettuce was involved primarily in the breakdown of sucrose^39^, thus SS activity in the present study was tested in sucrose degradation direction. Although not entirely consistent, the trend of SS activity in lettuce among different treatments was found to be very similar with that of AI. Compared with the consistent light environment in RB, the alternating light in R/B(4 h) and R/B(1 h) treatments reduced the activities of AI, NI and SS to some extent, while increased the activity of SPS. The integrated activity of sucrose degrading enzymes (invertase plus SS) in lettuce exposed to R treatment was markedly increased by 21–105% comparing with that in the other treatments. In addition, the strongest SPS activity accompanied with the highest content of sucrose was observed in plants treated with R/B(1 h).

### The contents of soluble sugar, starch and crude fiber

**Figure 6:**
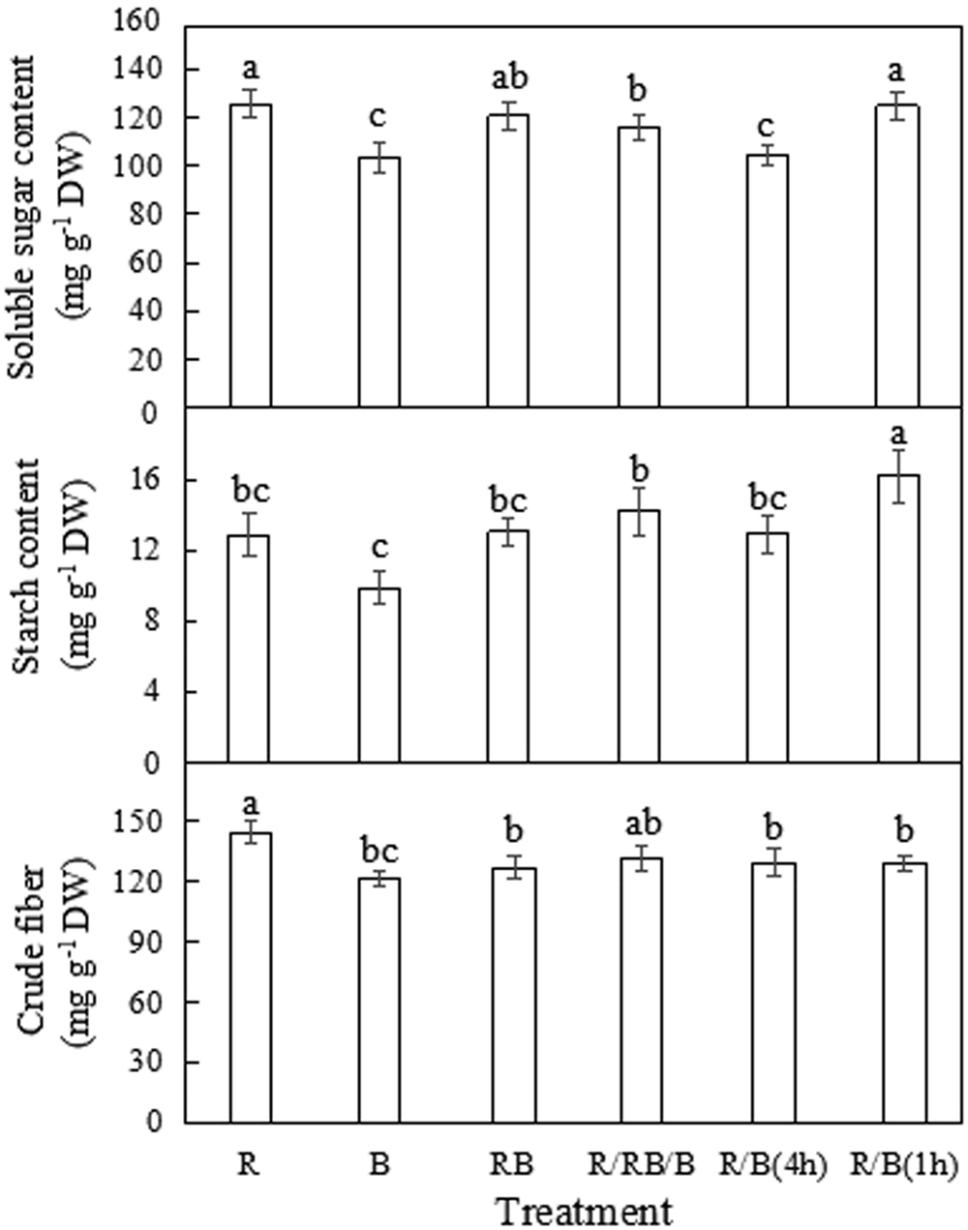
The contents of soluble sugar, starch and crude fiber of lettuce (at harvest) planted in varied light treatments. Lower-case letters with the same parameter indicate significant difference at the 0.05 level by Tukey’s test, n = 3. The bars indicate the standard errors. R: monochromatic red light for 16 h over a 16-hour photoperiod; B: monochromatic blue light for 16 h over a 16-hour photoperiod; RB: simultaneous red and blue light for 8 h over a 24-hour period; R/RB/B: light switching from monochromatic red (lasted for 4 h) to simultaneous red and blue (lasted for 4 h) and then to monochromatic blue (lasted for 4 h) over a 24-hour period; R/B(4 h): alternating red and blue light with an interval of 4 h over a 16-hour photoperiod; R/B(1 h): alternating red and blue light with an interval of 1 h over a 16-hour photoperiod.

As shown in Fig. 6, monochromatic R significantly increased crude fiber content by 24%, while monochromatic B significantly decreased the soluble sugar content by approximately 14% compared with the control. On the whole, monochromatic R accelerated carbohydrate accumulation in lettuce compared with the other lighting modes in the study, while monochromatic B performed the opposite function. Compared with the consistent light environment in RB, starch content in lettuce treated with R/B(1 h) was significantly increased by approximately 17%, but no significant difference was observed in soluble sugar and crude fiber content; R/B(4 h) significantly decreased the soluble sugar content but resulted in no significant difference in starch or crude fiber contents. It indicated that impacts of alternating lighting modes on carbohydrate accumulations were related to the alternating intervals and the type of carbohydrate. Additionally, no significant difference in carbohydrate content was caused by the dynamic light environment in R/RB/B treatment compared with the control.

## Discussion

Plants sense and respond to specific light wavelengths via photoreceptors^40^. Five photosensory systems have been identified so far, including phytochromes (phys), cryptochromes (crys), phototropins (phots), zeitlupe family (ztl, fkf1and lkp2) and UV Resistance locus 8 (UVR8)^41–44^. Five phytochromes (phyA through phyE) act as red/far-red light receptors, while three cryptochromes (cry1, cry2, cry3), two phototropins (phot1, phot2) and three zeitlupes (ztl, fkf1and lkp2) act as blue/ultraviolet light receptors^45–48^. Several photoreceptors are always simultaneously activated by nature light, the signal conduction performed by different photoreceptors is not independent but interferes with or depends on each other, and the relationship among photoreceptors may be also related to the light environment and specific plant physiological activities. For example, Casal^49^ reported that the actions of phyB and cry1 are synergistic under short photoperiods of simultaneous R and B, but are independent under continuous exposure to the same light; phot1 and phot2 are independent under blue light of low intensity (<1 mmol·m^−2^·s^−1^), but are synergistic under blue light of high intensity (>1 mmol·m^−2^·s^−1^)^50^. PhyB and cry2 are antagonistic in the induction of flowering and leaf flattering^41,51^, while are synergetic in seedling de-etiolation and the shade avoidance syndrome (SAS)^52,53^.

That is to say, there may be the cross talk as well as synergy between the photoreceptor signaling pathways of R and B, which are independent in some cases but are interactive in other cases. It was reported that constant illumination with B alone might have negative effects such as reduced Pn in many species due to impaired mesophyll conductance and chloroplast avoidance responses^54,55^. In the present study, although lettuce with monochromatic B demonstrated the lowest shoot biomass, when red light followed with an interval of 1 hour, the fresh and dry weighs of lettuce shoot were significantly increased by 51.2% and 140%, respectively. As regards to monochromatic R, although red light had been reported to have great positive effects on biomass accumulation, when followed with blue light at an interval of 1 hour, the fresh and dry weigh of lettuce shoot were also significantly increased by 12.4% and 16.3%, respectively. Besides, we detected that the contents of pigment, sucrose, soluble sugar in lettuce and TSI of lettuce were all the highest (or not significantly different from the maximum value) under R/B(1 h) compared with the other treatments. Strictly speaking, in R/B(1 h) treatment, red and blue light did not appear together even in any instant, thus, it is possible that monochromatic R or B can fully function without negative effects as long as a different light follows with a proper interval (e.g., from red to blue), which may be a possible explanation for the benefit generated by R/B(1 h) treatment.

As mentioned before, R and B are efficiently absorbed by chlorophyll, and have the largest impacts on various physiological activities of plants. Simultaneous R and B with proper ratio has been reported to be the best lighting strategy for most plants^56–58^. However, results in the present study showed that R and B did not need to be supplied to plants at the same time, on the contrary, alternating light of R and B with an interval of 1 h resulted in better yield and taste. The relationship between red and blue light in the process of action or in the photo-reaction process by light receptors of plants may not be instantaneous or transient, but rhythmed and accumulative. It is noteworthy that the daily light integral of R or B as well as the electric consumption were all the same among the treatment RB, R/RB/B, R/B(4 h) and R/B(1 h). Therefore, better results in treatment R/B(1 h) actually enhanced the electric use efficiency (EUE) and light use efficiency (LUE), and the results will be of great significance in practical production. In contrast, negative effects such as low shoot biomass, soluble sugar content and TSI was resulted by R/B(4 h) treatment, indicating that the interval between red and blue light largely influenced the relationship between red and blue light during the signal and response pathway.

Chen^38^ reported that lettuce under monochromatic R was fragile and thin at seedling stage, but plants gradually developed normally and became vigorous, and by harvest, the fresh weight of lettuce shoot treated with monochromatic R was respectively 46% and 92% higher than that treated with FL and RB (R:B = 1:1) treatments. It indicated that red light accelerated biomass accumulation mainly in the late growth stage rather than seedling stage. Similarly, in the present study, we found that the fresh weight of lettuce grown under R treatment was second only to that under R/B(1 h) treatment. Meanwhile, the highest (or not significantly different from the maximum value) contents of fructose, glucose, soluble sugar and TSI were observed in lettuce treated with R treatment, indicating that although not the optimal lighting strategy, monochromatic R could satisfy the growth of lettuce throughout the whole growth period. In a word, if only monochromatic light is allowed or available during lettuce cultivation, red light may be the only light quality that can basically meet the growth, yield and quality requirements of lettuce.

The enhanced hexose/sucrose ratio has been reported to be accompanied by the increased activities of SS (cleavage) or AI^13^. Meanwhile, high SPS activity and low activities of AI, NI and SS (cleavage) have been reported to result in the predominant accumulation of sucrose in some crops^26,59^. Similar results for sugar accumulation and sugar metabolism related enzymes were observed in the current study. Results showed that the highest hexose content together with the strongest integrated activity of sucrose degrading enzymes (SS plus invertase) were both detected in plants treated with monochromatic R. Additionally, the highest sucrose content accompanied with the strongest SPS activity were observed in lettuce treated with R/B(1 h). It indicated that different light environment might affect sugar compositions and the sweetness of lettuce via regulating the activities of enzymes involved in sucrose metabolism.

The carbohydrate contents and compositions determine both the flavor and the quality of vegetables. It was reported that higher soluble sugar proportion and lower crude fiber proportion in total carbohydrate resulted in better taste of lettuce^9,10^. In the study, lettuce treated with R/B(1 h) demonstrated the highest shoot biomass, meanwhile, the soluble sugar content and TSI of lettuce were not significantly different from the maximum value observed in lettuce treated with R treatment. Additionally, the crude fiber content of lettuce in R/B(1 h) treatment was also not significantly different from the minimum value observed in lettuce treated with B treatment. Therefore, in terms of lettuce yield, taste and energy use efficiency, dynamic light in R/B(1 h) treatment is the optimal lighting strategy in the present study.

## Conclusion

Alternating red and blue light with an interval of 1 h enhanced the accumulation of biomass, sucrose and starch in lettuce, and also promoted EUE and LUE. In terms of yield, taste and energy use efficiency, R/B(1 h) is the optimal lighting strategy in the present study. Compared with plants treated with RB, the fresh weight and the contents of fructose, glucose, crude fiber and TSI in lettuce were significantly enhanced by monochromatic R treatment. Although not the optimal lighting strategy, monochromatic R could basically meet the growth, yield and quality requirements of lettuce. Different irradiation modes might influence sugar compositions in lettuce via regulating the activities of sucrose metabolism enzymes such as SS, SPS, AI and NI.

## Data Availability

The data used to support the findings of this study are included within the article.
